# The Diseases of Children in Classical Times

**Published:** 1907-08-10

**Authors:** 


### The Evolution of Medicine.

### THE DISEASES OF CHILDREN IN CLASSICAL TIMES.

We know but little of children and of their position in the house in classical times. Homer gives us a few pictures, and there is plenty of information as to their education. But we have only a fragmentary knowledge of the lives they led, of the games they played, and of the illnesses from which they suffered.

### Pediatrics in Grecian Times.

Dr. Séguin (*Thèse de Paris*, No. 337, 1902. "La Médecine infantile chez les Grecs et les Romains") has collected many scattered references from classical authors in reference to the various diseases of children, and has founded upon them his thesis for the degree of M.D. Crates, the poet, as early as B.C. 449, mentions a hospital or sanatorium near the sea, though he does not say that it was built especially to accommodate children. A story told by Herodotus emphasises the fact that the Greeks reckoned by lunar months, and their children were consequently born in the tenth month of pregnancy. He says that Ariston, King of Sparta, having no children by two wives whom he had married in succession, took the wife of his friend Agetus, who bore him a son B.C. 560, before the ten months were accomplished. Ariston was troubled by the premature birth, and disputed his paternity, though he was afterwards convinced. His son, however, Demaratus, when he grew up questioned his mother, who replied, "Know that some women are brought to bed at nine months, others at seven months, and it is not all who go to ten months, and Ariston, your father, was himself convinced of this matter." Aristotle is of the same opinion, remarking that children born before the seventh month cannot be made to survive; those born during the seventh month may live, though they are usually weakly, and should be wrapped in wool. But in Egypt eight months' children are quite viable, though in Greece very few live.

### The Hippocratic Teachings.

Hippocrates has some valuable information about children which is still serviceable. He tells us of mastoid disease when he says that "in children an acute pain in the ear with continued high fever is dangerous, for there is reason to fear that the child will become delirious and die. Young people succumb to this affection on the seventh day, or even sooner; but the discharge of white pus from the ear gives them a chance of recovery. The great Father of Medicine appears also to have recognised the form of meningitis, now called tuberculous, and he describes it in the following words: "In certain fevers children are attacked with convulsions if the fever be acute; they are costive, sleepless, and worried by nightmare; they groan and change colour, their face becoming yellow, livid, or red. Such symptoms most often affect children from infancy until they are seven years of age. Older children and adults are less likely to be convulsed in their feverish attacks." He believed, very rightly too in some cases, that "watery urine is the most deadly form of urine in children," for he had perhaps noticed the appearances associated with a very low specific gravity which sometimes accompanies hydronephrosis, the result of a tight phimosis or of other congenital obstruction to the outflow of urine. Hippocrates taught that calculi were formed from an alteration of the milk, which provoked a heating of the belly and bladder, so that the inflammatory urine led to the deposit of a stone. His advice, therefore, was to give children wine mixed with a great deal of water, for their veins were thus less likely to be burnt up and dried.

### Folk Medicine.

Horace had no great idea of the folk medicine of his time, for he says in his Satires (*Sat.* ii. 3. 288-296):

> "'Jupiter, ingentes qui das adimisque dolores,'
> Mater ait pueri menses jam quinque cubantis,
> "Frigida si puerum quartana reliquerit, illo
> Mane die, quo tu indicis jejunia nudus
> In Tiberi stabit.' Casus medicusve levarit
> Ægrum ex præcipiti : mater delira necabit
> In gelidâ fixum ripâ febrimque reducet.
> Quone malo mentem concussa? timore deorum."

"'O Jupiter, thou who givest and takest away great pains,' so vows the mother of a boy already five months an invalid, 'if the cold ague fit leave my child, on the morning of the day you impose a fast he shall stand naked in the Tiber.' Luck or the doctor raises the patient from the crisis, yet the crazy mother will kill him placed on the cold bank, and will bring back his fever. With what malady is her mind disordered?—with superstition."

### The Treatment of Children's Disorders by Celsus.

Celsus, like Hippocrates, gives several indications for the treatment of children. Amongst other things, he says that warm baths are better for children and old people, and that children should only take their wine well diluted. Celsus notices that children bear diarrhœa badly, but that pains in the hips and shoulders and palsies are favourably influenced by the youth of the patient. The treatment for intestinal worms is much the same as that in use to-day, for he recommends pomegranate. The directions are to eat plenty of garlic overnight and vomit; on the following day to drink, fasting, a draught made by boiling a small handful of pomegranate roots in three measures of water until it is reduced to a third of its bulk, and then adding a little nitre. The treatment of struma corresponds with much that is taught at the present time. Celsus knew that the disease pursued a very slow course, and advises that, whatever treatment be adopted, the body should be strengthened by exercise and good food until the scarring is complete.

